# A Systematic Review of the Role of Magnetic Resonance Imaging in the Diagnosis and Detection of Neurovascular Conflict in Patients With Trigeminal Neuralgia

**DOI:** 10.7759/cureus.44614

**Published:** 2023-09-03

**Authors:** Nikita Bora, Pratap Parihar, Nishant Raj, Neha Shetty, Bhagyasri Nunna

**Affiliations:** 1 Radiodiagnosis, Jawaharlal Nehru Medical College, Datta Meghe Institute of Higher Education and Research, Wardha, IND

**Keywords:** magnetic resonance imaging, microvascular decompression, neurovascular contact, root entry zone, trigeminal neuralgia

## Abstract

Trigeminal neuralgia (TN) is a debilitating disorder causing severe, episodic, unilateral stabbing facial pain disturbing enough to disrupt the activities of daily life. Classic TN is caused due to compression injury of the trigeminal nerve at the cistern segment caused by either an artery or a vein, referred to as neurovascular contact or conflict (NVC). Magnetic resonance imaging (MRI) has been the standard tool for the diagnosis of NVC. This study aimed to determine the incidence of NVC in TN, as identified by MRI, assess the various MRI grading patterns among patients with TN, and identify the vessels primarily involved in NVC. A systematic search of studies that used MRI for the diagnosis of TN in reference to NVC was conducted on DOAJ and PubMed/PubMed Central. Data were extracted and entered into a Microsoft Excel spreadsheet. The outcomes measured were the incidence of NVC as shown in MRI, vessels involved in NVC, and MRI grading patterns. We identified and selected 20 studies that fulfilled inclusion/exclusion criteria. In total, 1,436 patients were enrolled in all included studies. The type of MRI used was 1.5 T or 3 T MRI. The mean age of the patients varied from 49 to 63 years, with an equivalent male-to-female ratio. NVC was seen in 1,276 cases out of 1,436 cases (88.85%) of TN on the ipsilateral side, as shown by MRI. The vessels involved were arteries in 80-90% of the cases, followed by veins. Among the arteries, the superior cerebellar artery was the most common artery (80-90% of cases). The grades of NVC as assessed by MRI included grades I, II, and III with varied proportions in different studies. NVC is a common problem in TN, wherein there is compression at the nerve root entry zone, and it shows a strong predilection for the elderly population. MRI seems to be a novel imaging diagnostic investigation to identify NVC associated with TN. Moreover, NVC grading must be done with MRI so that it may help the surgeon in stratifying the patient’s treatment.

## Introduction and background

The trigeminal nerve (fifth cranial nerve) is a mixed sensorimotor nerve, with three sensory nuclei and one motor nuclei [1,2], It is most commonly susceptible to neuropathic pain, also known as trigeminal neuralgia (TN), that arises out of conflict in the sensory region of the nerve. The prevalence of TN has been reported to be up to 4-13 cases per 100,000 population, with a primary predilection for females and the age groups of the 40s, 50s, and 60s [2,3].

TN is a debilitating disorder due to severe, episodic, unilateral stabbing facial pain which may be triggered by a simple touch, cleaning teeth, washing, and shaving, disturbing enough to disrupt the daily activities of living of a person, leading to depression in some cases [1,4].

The causes of TN remain varied, and it has been categorized into the following two forms: (a) classical TN, where no cause or etiology can be established, and (b) secondary or symptomatic TN, which is primarily on account of visible anatomic pathologies such as tumors, injuries, multiple sclerosis, arachnoid cysts, and inflammation [2-5].

Among the two types of TN, classic TN is the most common type, seen in 80-90% of the cases, and is characterized by compression injury of the trigeminal nerve at the cistern segment which is at the nerve root entry zone (REZ). The impingement is caused commonly by an artery and less commonly by a vein, referred to as neurovascular contact or conflict (NVC). NVC may be just a simple contact of the artery/vein on the REZ region, or it may be a severe compression of the trigeminal nerve leading to nerve displacement or nerve atrophy. Primary vessels involved in causing NVC include the superior cerebellar artery, inferior and anterior cerebellar artery, or sometimes the basilar artery [2,6]. Notwithstanding, other central abnormalities or anatomical changes in the centers of pain in the brain must also be taken into account as the cause of classic TN in some cases [5].

In the cases of classic TN, identification of NVC becomes important as it may be surgically treated, providing complete relief to the patient [5]. For the diagnosis of NVC in classical TN, magnetic resonance imaging (MRI) has been a standard tool as it provides a much clearer view of the trigeminal nerve, its REZ, and the vessels that are impinging on the REZ with clear anatomical and vascular relationship [5]. This allows for a better surgical approach in the form of vascular decompression, microvascular decompression (MVD), or ablative procedures [7].

From the clinician’s point of view, it is also important to determine the various points of contact leading to NVC in the trigeminal nerve, for which imaging investigations (MRI) hold supreme importance. We cannot proceed with surgery without obtaining an MRI. Moreover, MRI also allows for the detection of secondary pathologies that may be present, such as tumors, demyelination, mass lesions, and infections, around the nerve which may lead to classic TN [1,5-7].

The literature has explored the accuracy of MRI in detecting NVC in symptomatic cases and asymptomatic cases of TN, and it was seen that MRI shows varied sensitivity (Sn) and specificity (Sp) for diagnosing NVC (despite its tremendous power to delineate anatomy). Moreover, NVC may not only be present in cases of classic TN, as it may also be present in healthy control populations without causing any symptoms. A systematic review by Antonini et al. [5], reported that for diagnosing NVC with the criteria of REZ contact, MRI had an Sn and Sp of 66% and 90%, respectively, for nerve atrophy, an Sn and Sp of 54% and 97%, respectively, for nerve dislocation, MRI had Sn and Sp of 39% and 97%, respectively, while for REZ contact + atrophy, MRI had an Sn and Sp of 52% and 100%, respectively. Hence, taking together different criteria for diagnosing NVC, MRI holds great specificity but its sensitivity falls short.

For further exploring the role of MRI in the detection of NVC (keeping in mind that not all patients with classic TN have NVC), we conducted this systematic review to determine (a) the incidence of NVC in TN, as identified by MRI, (b) the various MRI grading patterns among patients with TN, and (c) the vessels primarily involved in NVC.

## Review

Methodology

Literature Search

Keeping in mind the Preferred Reporting Items for Systematic Reviews and Meta-Analyses (PRISMA) guidelines, a systematic search of studies that used MRI for the diagnosis of TN in reference to NVC was conducted on two primary databases, namely, DOAJ and PubMed/PubMed Central. The data search was conducted by two primary authors and data extraction was done by another two authors. The keywords used for the data search were magnetic resonance imaging, neurovascular conflict OR neurovascular contact, trigeminal neuralgia, and these three keywords were separated by the boolean operator AND. The fifth author read the titles, abstracts, and full texts of the articles to determine if they were eligible based on the inclusion/exclusion criteria shown in Table 1.

**Table 1:** Inclusion and exclusion criteria.

Inclusion criteria	Exclusion criteria
Articles written in the English language.	Non-English-language articles
Articles published from 2009 until 2023	Articles published before 2009
Articles for which full text was freely downloadable	Articles for which full text was downloadable at a subscription
Articles where neurovascular conflict was detected based on MRI in patients with trigeminal neuralgia	Animal research studies
Observational/prospective cohort studies or case-control studies	Book chapter/editorials/systematic review and meta-analysis

Data Extraction

Data extraction was done by two primary authors, wherein after downloading all articles in the full-text pdf format, the articles were printed and data were extracted and entered into a Microsoft Excel spreadsheet under the columns of serial number, first author, year, place/location, time period, number of cases, MRI used, mean age of the patients, gender distribution, MRI findings which included Grade 1 n (%), Grade 2 n (%), and Grade 3 n (%), NVC n (%), and the type of vessel involved with its percentage.

Assessment of Risk of Bias

The ROBINS-I tool was used for assessing the risk of bias, wherein seven parameters were analyzed, which include measurement bias, selection bias, confounding bias, attrition bias, outcome reporting bias, and performance bias. Then, the overall risk was calculated and categorized into high, moderate, and low based on the scores, with low having a score of 0 to 1, moderate having a score of 2 to 5, and high having a score of 6 or more.

Outcomes

The outcomes measured were the incidence of NVC as shown on MRI, vessels involved in NVC, and MRI grading patterns.

Results

**Figure 1:**
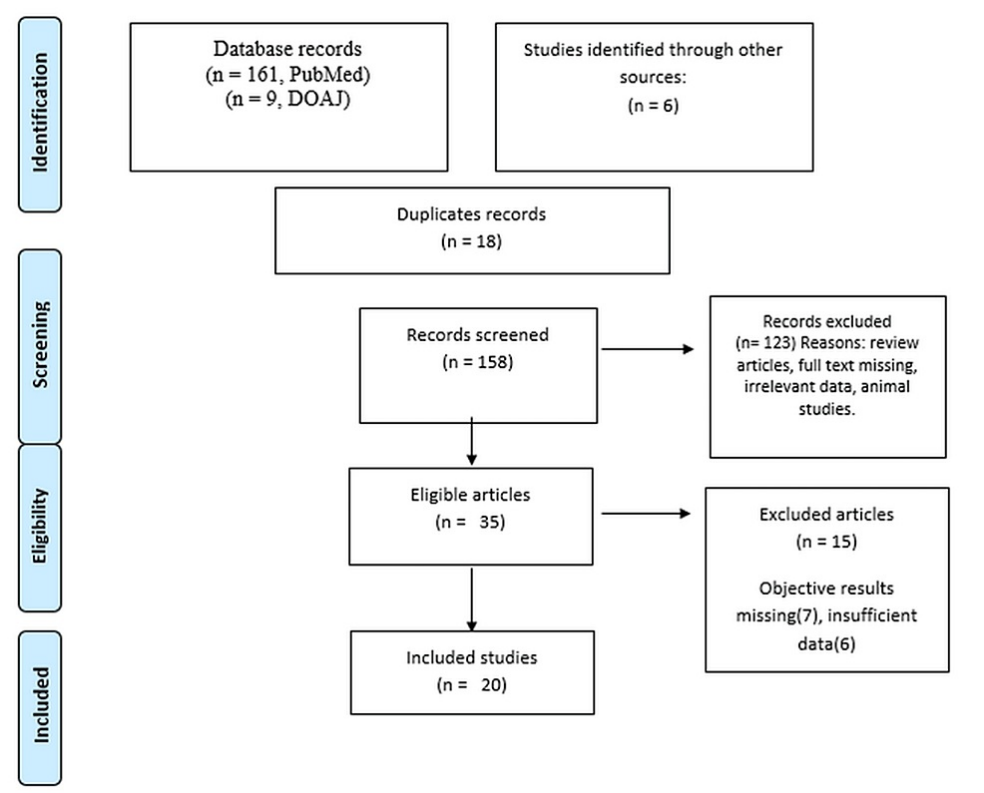
Preferred Reporting Items for Systematic Reviews and Meta-Analyses flowchart showing the search strategy.

On an extensive search of the primary databases, we found 161 articles in PubMed/ Pubmed Central, nine articles in DOAJ, and six articles from alternate sources such as sub-references from meta-analyses and previous studies. After excluding 18 duplicates, 158 articles were screened from the title and abstracts. Of these, 35 articles were found to be eligible whose full text was downloaded after excluding 123 articles according to the exclusion criteria. After reading 35 articles, 20 were selected for the systematic review while 15 were excluded as objective data were missing. Figure 1 shows the PRISMA flowchart for the search strategy.

For the systematic review, 20 studies were finalized, whose data were retrieved, as shown in Table 2.

**Table 2:** Study characteristics. AICA: anterior inferior cerebellar artery; CISS: constructive interference in steady state; FIESTA: fast imaging employing steady-state acquisition; SCA: superior cerebellar artery; VA: vertebral artery; BA: basilar artery; PCA: posterior cerebral artery

Author	Year	Place	Duration of study	N	MRI used	Mean age (years)	M:F	NVC n(%)	Vessel involved with %	MRI – Grades I, II, III
Chun-Cheng et al. [8]	2009	China	-	45	3T	55.67	55.5%/45.5%	40 (95.23%) of the 42 symptomatic nerves	Arterial involvement: 75%, vein: 20%, multivessel involvement: 5%	-
Lorenzoni et al. [9]	2009	Switzerland, Europe	4 years 9 months	100	1.5 T	-	-	93 (93%)	SCA: 76%, AICA: 11%, basilar artery: 2%	-
Miller et al. [10]	2009	Oregon, USA	9 months	30	3 T	54.8	43%/57%	17 (57%)	SCA in all cases	-
Peker et al. [11]	2009	Turkey		100	3T	49	44%/56%	9 2(92%)	Arteries: 86%, veins: 14%	-
Ni et al. [12]	2009	China	1 year	29	3T	58.2	42.42%/57.58%	27 (93%)	SCA: 37.9%, SCA+SPV: 17.24%	-
Leal et al. [13]	2010	France/Europe	3 years	91	1.5 T	-	-	80 (87.9%)	-	-
Leal et al. [14].	2010	France/Europe	2 years 4 months	100	1.5 T	61	46%/54%	91 (91%)	SCA: 61%, AICA: 10%, BA: 3%	Grade I: 12%, Grade II: 40%, and Grade III: 39%
Cha et al. [15]	2011	Korea, Asia	2 years 9 months	66	3 T	59	43.9%/56.1%	63 (95.5%)	SCA: 73.5%, petrosal vein: 10.2%, AICA: 8.2%	-
Vergani et al. [16].	2011	Tyne, UK	4 years	67	1.5 T	59.8	46.26%/53.74%	63 (94%)	-	-
Antonini et al. [5]	2014	Italy, Europe	1 and half year	24	1.5 T	63	45.83%/54.17%	21 (87.5%)	-	-
Maarbjerg et al. [17]	2015	Denmark (Europe)	1 year 7 months	135	3 T		56%/44%	71 (53%)	-	-
Rangaswamy et al. [2].	2016	India	1 year	75	1.5 T, 3D-CISS	52	58%/42%	45 (60%)	SCA: 48%, AICA: 12%	-
Mistry et al. [18]	2016	USA	Retrospective data 2004–2010	57	3T	55	22.8%/77.2%	47 (82.45%)	-	-
Ruiz-Juretschke et al. [19]	2019	Spain		100	3 T	59	41%/59%	142 (71%) out of 200 nerves	Venous compression: 66%, SCA: 28%, AICA: 2%	Grade I: 92.3%, Grade 2: 7%, and Grade 3 in 0.7% case
Wei et al. [20]	2020	China	3 years	146	3 T	56	46.57%/53.43%	143 (97.9%)	Arterial contact: 75.34%, arterial and venous contact: 15%, venous contact: 7.53%	-
Müller et al. [21]	2020	Germany	6 years	48	3T	59.63	66.67%/33.33%	39 (81.25%)	Arterial: 64.1%, venous: 23%	-
Vedaraju et al. [6]	2021	India	2 years	30	3 T, FIESTA C sequence	53	40%/60%	30 (100%)	SCA: 73%, AICA: 21%, PCA: 3%, VA: 3%	Grade I: 56.67% Grade II: 23.33% Grade III: 20%
Anwar et al. [3]	2022	India	2 years 1 month	76	1.5 T, FIESTA sequence	55.6	35.8%/64.2%	67 (88%)	SCA: 77%, AICA: 23%	
Singhal et al. [22]	2022	Australia	7 years	68		62	-	57 (83.8%)	-	-
Jiang et al. [23]	2022	China	-	49	3T	53.1	42.85%/57.15%	48 (98%)	-	-

Study Characteristics

Various studies have been published on the role of MRI in diagnosing NVC from 2009 to 2023 in different countries such as China, Europe, Switzerland, the United States, Turkey, Korea, Italy, Denmark, Spain, and Germany. The duration of the studies varied from nine months to seven years. The total number of patients enrolled in all studies was 1,436. The type of MRI used was 1.5 T or 3 T MRI. The mean age of the patients varied from 49 to 63 years, with an equivalent male-to-female ratio.

Study Outcomes

NVC was seen in 1,276 cases out of 1,436 cases (88.85%) of TN on the ipsilateral side, as shown by MRI. The vessels involved were arteries in 80-90% of the cases, followed by veins. Among the arteries, the superior cerebellar artery was the most common, followed by others which included the anterior inferior cerebellar artery, vertebral artery, and basilar artery in decreasing order. The grades of NVC as assessed by MRI included Grades I, II, and III with varied proportions in different studies. Ruiz-Juretschke et al. [19], and Vedaraju et al. [6] reported Grade I as the most common as seen in 92.3% and 56.67% of cases while Leal et al. [14], reported Grades II and III as common grades.

Risk of Bias

In five studies, the risk of bias was low [2,9,12,16,21], and in the remaining 15 studies, moderate risk was present [3,5,6,8,10,11,13-15,17-20,22,23] (Table 3).

**Table 3:** Risk of bias assessment. L: low; M: moderate

Name of the author	Confounding bias	Selection bias	Measurement of interventions bias	Intended interventions (performance bias)	Attrition bias	Measurement bias	Outcome reporting bias	Overall Risk of bias
Chun-Cheng et al. [8]	-	+	+	+	-	-	+	M
Lorenzoni et al. [9]	-	-	-	-	-	-	-	L
Miller et al. [10]	-	+	-	-	+	-	-	M
Peker et al. [11]	-	+	-	-	+	-	-	M
Ni et al. [12]	-	-	-	-	-	-	-	L
Leal et al. [13]	+	-	+	+	-	-	+	M
Leal et al. [14]	-	+	-	-	+	-	-	M
Cha et al. [15]	+	-	-	+	+	-	+	M
Vergani et al. [16]	-	-	-	-	-	-	-	L
Antonini et al. [5]	-	-	+	+	-	-	+	M
Maarbjerg et al. [17]	+	-	-	+	+	-	+	M
Rangaswamy et al. [2]	-	-	-	-	-	+	-	L
Mistry et al. [18]	-	+	-	-	+	-	-	M
Ruiz-Juretschke et al. [19]	-	+	-	-	+	-	-	M
Wei et al. [20]	-	-	+	+	-	-	+	M
Müller et al. [21]	-	-	-	-	-	-	-	L
Vedaraju et al. [6]	-	+	+	-	-	-	+	M
Anwar et al. [3]	+	-	-	+	+	-	+	M
Singhal et al. [22]	+	-	+	-	-	-	+	M
Jiang et al. [23]	-	+	-	-	+	-	-	M

Discussion

The present systematic review holds importance as it analyzes the presence of NVC as per MRI worldwide. MRI has become a basic investigative norm for patients presenting with TN not only for detecting NVC but also for identifying further secondary pathologies. The presence of NVC was seen in 80-100% of the cases in various studies [3,5,6,8,9,11-16,18,20-23]. However, few studies have reported a lower incidence of NVC in the range of 50-80% [2,10,17,19].

The varied findings among different studies may be because of different MRI sequences used. Anwar et al. [3] used 1.5 T with FIESTA sequence and Vedareju et al. [6] used 3 T with FIESTA sequence, while Rangaswamy et al. [2] used 1.5 Tesla with 3D CISS sequence rather than FIESTA sequence. It was presumed that the CISS sequence had a superior resolution in terms of contrast and spatial arrangement for demonstrating the NVC. The trigeminal nerve was identified per its anatomical course from Meckel’s case at the posterior end to the pre-pontine cistern. As per the 3D constructive image, the arteries were identified as they were traced back to their origin from the basilar artery. As for the 3D FIESTA sequence, it was said that whether it is the vessel or nerve, both of them appear black and are enclosed by high cerebrospinal fluid (CSF). If there was any contact between the vessel and the nerve, there was a change in the signal intensity which was presumed to be better identified by the 3D FIESTA sequence [6]. Other MRI protocols include the 3D time of light, magnetic resonance angiography, and 3D TOFF MRA, and there is high-resolution T2-weighted imaging or there may be a combination of the two and there may be 3D multi-model image fusion as well.

The major arteries identified as the root cause of NVC were numerous, with SCA being the most common [2,3,6,9,10,12,14,15,19]. In the review, we observed that the grading was done by only a few studies [6,14,19]. The grading was based on the description that Grade 1 involves only the contact without the interposing CSF layer. Grade 2 involves nerve root deviation, and Grade 3 involves nerve root indentation [6]. This grading helps in a better approach to surgery, and it is proposed that future studies should do the grading and report so that it can be used for the outcomes of the surgeries and planning the surgeries.

Despite MRI showing true evidence of NVC with elaborated MRI sequences helping in the diagnosis of NVC, the gold standard for NVC still remains the intraoperative confirmation, and on this front, other imaging studies lack the power to diagnose NVC.

Strengths

This systematic review covers all published studies worldwide that used different sequences of MRI for diagnosing NVC in TN, and thus it represents multicentric data without any significant bias in the reporting of the performance of MRI for diagnosing and determining the incidence of NVC in patients with TN.

Limitations

The systematic review must be read under the preview of certain limitations. First, a meta-analysis of the studies was not done. Second, the data from case-control comparative studies were not reported, and no sensitivity and specificity of MRI for diagnosing cases with NVC in TN patients and in asymptomatic cases were reported. Third, not all studies included gold standard confirmation with intraoperative management, thereby limiting the power of the studies. Fourth, the review did not include articles that were not open-access.

## Conclusions

NVC is a common problem (88.85%) among cases of TN, wherein there is compression at the nerve REZ, and it shows a strong predilection for the middle-aged to the elderly population. The most common artery involved is SCA (85% of cases). MRI seems to be a novel imaging diagnostic investigation to identify NVC associated with TN with the presentation of patients with different grades of compression ranging from Grades I to III However, as MRI may not be 100% sensitive, the MRI reports (whether negative or positive) must be correlated with clinical symptoms for identifying patients to proceed with the surgery. Moreover, NVC grading must be done with MRI so that it may help the surgeon to stratify the patient’s treatment. The advancing MRI sequences hold a role in the future with upcoming FIESTA sequencing and 3D CISS sequence which give a clearer spatial arrangement of the trigeminal nerve and the vessels that may compress the REZ.
